# Conservation genomic study of *Hopea hainanensis* (Dipterocarpaceae), an endangered tree with extremely small populations on Hainan Island, China

**DOI:** 10.3389/fpls.2024.1442807

**Published:** 2024-09-04

**Authors:** Liang Tang, Jun-qiao Long, Hai-ying Wang, Chao-kang Rao, Wen-xing Long, Li Yan, Yong-bo Liu

**Affiliations:** ^1^ International Joint Center for Terrestrial Biodiversity around the South China Sea of Hainan Province, Hainan University, Haikou, China; ^2^ School of Ecology, Hainan University, Haikou, China; ^3^ Haikou Marine Geological Survey Center, China Geological Survey, Haikou, China; ^4^ School of Tropical Agriculture and Forestry, Hainan University, Haikou, China; ^5^ State Environmental Protection Key Laboratory of Regional Eco-Process and Function Assessment, Chinese Research Academy of Environmental Sciences, Beijing, China

**Keywords:** *Hopea hainanensis* Merrill & Chun, conservation genomics, plant species with extremely small populations, population decline, reduced-representation genome sequencing

## Abstract

**Introduction:**

*Hopea hainanensis* Merrill & Chun is considered a keystone and indicator species in the tropical lowland rainforests of Hainan Island. Owing to its high-quality timber, *H. hainanensis* has been heavily exploited, leading to its classification as a first-class national protected plant in China and a plant species with extremely small populations (PSESPs).

**Methods:**

This study analyzed genome-wide single nucleotide polymorphisms obtained through restriction site-associated DNA sequencing from 78 adult trees across 10 *H. hainanensis* populations on Hainan Island.

**Results and discussion:**

The nucleotide diversity of the sampled populations ranged from 0.00096 to 0.00138, which is lower than that observed in several other PSESPs and endangered tree species. Bayesian unsupervised clustering, principal component analysis, and neighbor-joining tree reconstruction identified three to five genetic clusters in *H. hainanensis*, most of which were geographically widespread and shared by multiple populations. Demographic history analysis based on pooled samples indicated that the decline in the *H. hainanensis* population began approximately 20,000 years ago, starting from an ancestral population size of approximately 10,000 individuals. The reduction in population size accelerated approximately 4,000 years ago and has continued to the present, resulting in a severely reduced population on Hainan Island. Intensified genetic drift in small and isolated *H. hainanensis* populations may contribute to moderate differentiation between some of them, as revealed by pairwise *F*_st_. In conclusion, our conservation genomic study confirms a severe population decline and an extremely low level of nucleotide variation in *H. hainanensis* on Hainan Island. These findings provide critical insights for the sustainable management and genetic restoration of *H. hainanensis* on Hainan Island.

## 1 Introduction

Tropical rainforests play crucial roles in local economies and ecosystem services (Corlett and Primack, 2011). They produce a variety of biomass materials, sustain extremely high biodiversity, and are key in carbon sequestration, temperature regulation, watershed services, and climate change mitigation (Montagnini and Jordan, 2005; Corlett and Primack, 2011). Asian tropical rainforests flourish in the Malay Peninsula, Sumatra, Borneo, Java, New Guinea, and wetter region of the Philippine Islands, extending north through the Indochina Peninsula to the southern parts of Yunnan, Guangxi, Guangdong, and Taiwan Provinces, as well as Hainan Island in China (Hu and Li, 1992). A single family, the Dipterocarpaceae, dominates the lowland rainforests in Asia, with the species diversity, richness, and cross-sectional area at breast height being most prominent in these forests (Ghazoul, 2016). Trees of the Dipterocarpaceae are primarily large canopy trees, accounting for more than 50% of all canopy species, with many exceeding 50 m in height. No other tropical forests have such a high proportion of dominant species in a single family (Ghazoul, 2016). Research on the phylogeny, divergence time, historical biogeography, and population genetics of Dipterocarpaceae will facilitate a better understanding of the assembly, evolution, and adaptation of Asian tropical rainforest communities (Ashton, 1988; Ghazoul, 2016).

Hainan Island is located on the northern edge of tropical Asia, where the climate is heavily influenced by the Asian monsoon. The temperature and precipitation on Hainan Island are markedly different from those in Southeast Asia near the equator (Hu and Li, 1992; Zhu, 1993). The species diversity and abundance of dipterocarps on Hainan Island are much less prominent than those in Southeast Asia (Hu and Li, 1992). In fact, there are only three species of Dipterocarpaceae on the island: *Vatica mangachapoi* Blanco, *Hopea hainanensis* Merrill & Chun, and *H*. *reticulata* Tardieu (Li et al., 2007; Xing et al., 2012). *V. mangachapoi* is the dominant species and is widely distributed in the lowland rainforests of Hainan Island (Hu and Li, 1992; Li et al., 2006). However, *H*. *reticulata* is confined to the Ganza Ridge, and its fruits are wingless, falling directly to the ground after maturity (Hu et al., 2020). *H*. *hainanensis*, a species with an extremely small population, is rare in the lowland rainforests. Two sepals in the flower of this species continue to grow and develop into wing-like lobes in the fruits, aiding in seed dispersal (Li et al., 2007). It is worth noting that *H*. *hainanensis* serves as an indicator of the development of tropical rainforests on Hainan Island and has the highest importance value in the lowland rainforest communities (Hu, 1983). Additionally, the timber from *H*. *hainanensis* is highly valued in Hainan due to its high density, hardness, and corrosion resistance (Xie and Huang, 1990). As a result, *H*. *hainanensis* has been excessively logged, and mature trees are now very rare, with fewer than 250 remaining (Information system of Chinese Rare and Endangered Plants: http://www.iplant.cn/bhzw/info/985). In addition to Hainan Island, the occurrence of this species has been recorded in a few locations in Vietnam (Li et al., 2007). Owing to the extremely small size of its natural populations and its ecological significance in the tropical forests of Hainan Island, *H*. *hainanensis* has been listed as a national first-class protected plant in China (Yang et al., 2016; Lu et al., 2020).

Plant species with extremely small populations (PSESPs) refer to endangered plants that are at risk of extinction without protection because their wild population sizes are smaller than the minimum viable population size (Ren et al., 2012; Ma et al., 2013; Zang et al., 2016). In the past 10 years, increasing attention has been paid to exploring the possible causes of population declines and the sustainable management of PSESPs. Yao et al. (2021) suggested that reduced fertility and consequent difficulty in regeneration, loss of genetic diversity and compromised adaptation potential in small populations, human disturbance, natural disasters, and global climate change contribute to the occurrence of PSESPs. Zang et al. (2016) proposed a scheme for the maintenance and restoration of PSESPs, which includes *in situ* conservation and habitat restoration, *ex situ* conservation, seedling propagation and field planting, and genetic evaluation of germplasm resources.

The proposal of PSESPs and efforts to protect them have facilitated the rescue of critically endangered plant species in China (Sun et al., 2019). Conservation genetic studies have been carried out for some PSESPs in Yunnan Province, China. Using a combined strategy of *de novo* genome sequencing and whole-genome resequencing, the nucleotide diversity and demographic history of two PSESPs, *Rhododendron griersonianum* and *Acer yangbiense*, were assessed (Ma et al., 2021, 2022). Research on another PSESP, *H*. *hainanensis*, has mainly focused on its population ecology, seed germination, and seedling growth (Wen et al., 2002; Zhang et al., 2019; Lu et al., 2020). Previous studies have found that adult *H*. *hainanensis* trees can bear a large number of fruits that easily germinate and form a large number of seedlings after falling to the forest ground. However, few of these seedlings grow into saplings or young trees, indicating a severe recruitment constraint in the natural populations of *H*. *hainanensis* on Hainan Island (Lu et al., 2020; Luo et al., 2023). The shady environment under the canopy and fierce competition between the seedlings result in the death of almost all the seedlings. The development of the seedings is also affected by other habitat factors, such as slope, soil moisture and nutrients, and distance from mother trees (Pei et al., 2015; Lu et al., 2020). These studies have provided valuable insights into the *in situ* conservation and restoration of *H*. *hainanensis*.

Knowledge of the level and pattern of genetic variation in endangered species can be used to infer the causes of population declines, define conservation units, identify populations needing urgent protection, and design sampling schemes for *ex situ* conservation (Chen et al., 2002; DeSalle and Amato, 2004; Benestan et al., 2016; Li et al., 2020). By genotyping 12 polymorphic microsatellite markers, Wang et al. (2020) assessed the genetic variation patterns of *H*. *hainanensis* on Hainan Island. It was found that there is a lack of low-frequency alleles in the wild population of *H*. *hainanensis*, suggesting a potential recent bottleneck in this species. However, details of its demographical history, such as the timing and intensity of the bottleneck, have not been fully determined. It remains unclear whether the bottleneck was induced by paleoclimate changes or human disturbance or both. Additionally, there are inherent limitations in the application of microsatellite markers in population genetic analyses (Schlotterer, 2004; Putman and Carbone, 2014). Fortunately, genome-wide single nucleotide polymorphisms (SNPs) can be detected and genotyped using next-generation sequencing in a high-throughput and cost-effective manner (Davey et al., 2011). For non-model species without reference genomes, restriction site-associated DNA sequencing (RADseq) has been routinely performed to discover a large number of SNPs, enabling ecological, evolutionary, and conservation genetics studies (Davey and Blaxter, 2010; Andrews et al., 2016; Parchman et al., 2018).

In the present study, genome-wide SNPs were genotyped for *H*. *hainanensis* using RADseq, and conservation genomic research was conducted to shed light on the sustainable management of this dipterocarp species on the northern edge of the Asian tropics. The aims of this study were to determine the following: (1) the level of nucleotide diversity in *H*. *hainanensis* on Hainan Island—is it higher or lower compared with other endangered trees? (2) The geographic distribution of genetic variation—how is the nucleotide variation in this species structured on the island? (3) The demographic history of *H*. *hainanensis* and possible causes of its population decline.

## 2 Materials and methods

### 2.1 Population sampling

We collected 10 populations of *H*. *hainanensis* on Hainan Island, including most of the known locations of this species. Among them, nine populations were sampled from the Hainan Tropical Rainforest National Park, and one was collected from Baolong Forest Farm in Sanya City. Detailed information on population codes, sample sizes, and geographic locations (latitude and longitude) is listed in **Table 1**. Leaf samples were collected from adult trees with a diameter at breast height greater than 0.1 m. All sampled trees were separated by a distance of at least 10 m. Young disease-free leaves were collected and dried with silica gel immediately. After completely drying, the leaves were stored in a –nore refrigerator for later use.

**Table 1:** Geographic origin, population size, and nucleotide diversity of *Hopea hainanensis* on Hainan Island.

Population code	Sample size	Collection locality	Geographic coordinates	*H*_o_	*H*_e_	*F*_is_	π
FJ	9	Fanjia natural reserve	19.2722°N, 109.6150°E	0.09910	0.20210	0.30837	0.00129
LM	8	Limu Mountain	19.1909°N, 109.7417°E	0.23612	0.22192	0.01669	0.00110
BW	8	Bawang Mountain	19.0982°N, 109.1313°E	0.15116	0.19720	0.14599	0.00125
QW	7	Qiwang Mountain	18.9388°N, 109.4468°E	0.26692	0.22875	-0.01922	0.00101
JX	8	Jiaxi natural reserve	18.8429°N, 109.1662°E	0.24881	0.22565	-0.00205	0.00112
JF	8	Jianfeng Mountain	18.7422°N, 108.9902°E	0.16262	0.23548	0.22844	0.00134
KF	7	Kafa Mountain	18.6988°N, 109.3303°E	0.21524	0.19869	0.02133	0.00096
MR	7	Maorui forestry station	18.6724°N, 109.4116°E	0.10925	0.22807	0.34535	0.00138
DL	8	Diaoluo Mountain	18.6961°N, 109.8839°E	0.13987	0.18339	0.13718	0.00107
BL	8	Baolong forestry station	18.4855°N, 109.4385°E	0.22960	0.23223	0.06851	0.00114
Total	78			0.20414	0.25982	0.26431	0.00119

H_o_, observed heterozygosity; H_e_, expected heterozygosity; F_is_, inbreeding coefficient; πo nucleotide diversity.

### 2.2 Restriction site-associated DNA sequencing and data analyses

Total genomic DNA was extracted from the silica gel-dried leaves using a Qiagen plant genome extraction kit (Qiagen, Shanghai, China). The concentration and quality of the DNA were determined using a Qubit 3.0 Fluorometer. After appropriate dilution, genomic DNA was double digested with EcoRI and MseI. The digested fragments were cleaned and subsequently quantified using agarose gel electrophoresis, then ligated to EcoRI and MseI adapters containing sample specific barcodes. After ligation, individually barcoded samples were size-selected (350-sel bp) using agarose gel (Omega kit) and purified. The resulting fragments were further amplified by PCR to the desired concentration and sequenced on the HiSeq X Ten platform (Illumina) with PE 150 mode.

Raw reads generated by RADseq were analyzed using the Stacks version 2 package (Rochette et al., 2019). The script ‘*process_radtags.pl*’ was executed to demultiplex the raw sequencing reads and remove low quality data. Then, the ‘*denovo_map.pl*’ program was run to assemble loci and call SNPs without a reference genome using the demultiplexed reads. The number of mismatches allowed between the two alleles of an individual was controlled by the parameter *M*, and its optimal value was determined according to the method suggested by Rochette and Catchen (2017). SNPs were filtered using the ‘*populations*’ program in Stacks 2. The filters included the following: 1) at least 50% of the studied populations and at least 60% of the individuals in each population must be present to process a locus; 2) the minimum minor allele frequency at a locus is 0.05; and 3) only one random SNP per locus is retained for the analyses of population structure and historical demography. A variant call format (VCF) file and an input file for STRUCTURE version 2.3.4 (Pritchard et al., 2000) were also generated by ‘*populations*’ and used for subsequent data analysis.

### 2.3 Population genetic data analyses

Nucleotide diversity (ive observed and expected heterozygosity (*H*_o_ and *H*_e_), inbreeding coefficient (*F*_is_), and genetic differentiation between populations (*F*_st_) were estimated by the ‘*populations*’ program in the Stacks 2 package. An individual-based *p*-distance matrix was calculated using the software VCF2Dis (https://github.com/BGI-shenzhen/VCF2Dis), then a neighbor-joining (NJ) tree was reconstructed using the ‘*fneighbor*’ program and the bootstrapping consensus tree was inferred by the ‘*fconsense*’ program (Rice et al., 2000). Population structure was analyzed using the model-based clustering method implemented in the software STRUCTURE version 2.3.4 (Pritchard et al., 2000). The number of populations (*K*) varied from 1 to 12, and for each value of *K*, 10 independent replicates were run with 100,000 burn-in iterations followed by 1,000,000 Markov chain Monte Carlo simulations. A mixed model with correlated allele frequencies was used (Falush et al., 2003). STRUCTURE Harvester was used to determine the most likely number of populations (*K*) following the procedure described by Evanno et al. (2005), and a graphical representation of the clustering analysis was generated by the web-based software StructureSelector (Li and Liu, 2018). Principal component analysis was performed using the GCTA package with default settings, and the first and second principal components were selected for plotting (Yang et al., 2011).

To assess whether populations followed a pattern of isolation by distance, we plotted the pairwise genetic differentiation between populations, estimated by Wrightte *F*_st_, against the geographical distance using the Mantel test (Mantel, 1967) as implemented in Arlequin 3.5 (Excoffier and Lischer, 2010). The significance of the test was determined by 9,999 random permutations. An analysis of molecular variance (AMOVA), implemented in Arlequin 3.5, was conducted to detect population genetic differentiation at inter-population and intra-population levels. The significance of the *F*-statistics was determined by 9,999 random permutations.

A folded SNP frequency spectrum (SFS) was generated by the Python script *easySFS* (https://github.com/isaacovercast/easySFS). To utilize SNPs with partially missing data, the “down projection method” implemented in *easySFS* was used to determine the optimal projection value for the input data. The demographic history of *H*. *hainanensis* on Hainan Island was inferred based on the folded SFS using the software Stairway Plot 2, a model-free method that does not require whole-genome sequencing data or a reference genome (Liu and Fu, 2015; 2020). A mutation rate was required to convert time into the unit of years. Based on node times and the nucleotide sequences reported in Heckenhauer et al. (2017), the averaged mutation rate of the genus *Hopea* was estimated to be 2.13e−9 at per site per year. Field records indicate that *H*. *hainanensis* begins to bloom at approximately 30 years old. Therefore, the generation time of this species was set to 20, 30, and 40 years per generation to account for the potential variation in the timing of first flowering. The 10 sampled populations were pooled for Stairway Plot 2 analysis.

## 3 Results

### 3.1 Nucleotide diversity of *H*. *hainanensis* on Hainan Island

After the removal of low-quality reads, 247,235,540 clean reads remained, with an average of 3,169,686 reads per sample. The numbers of reads and nucleotides for each individual are listed in **Supplementary Table S1**. The filtered sequencing data have been deposited in the National Center for Biotechnology Information (NCBI) Sequence Read Archive (SRA) under accession number PRJNA1083891. The best fit value of the assembly parameter *M* was determined to be 3, following the method proposed by Rochette and Catchen (2017). Using this value of *M*, 6,017 loci were kept after filtering, of which 3,411 were found to contain 7,804 SNPs. The filtered SNPs were saved in a VCF format file for subsequent data analysis. The observed and expected heterozygosity, inbreeding coefficient, and nucleotide diversity were calculated using the ‘*populations*’ program. The observed heterozygosity ranged from 0.09910 (FJ) to 0.26692 (QW), and the expected heterozygosity ranged from 0.19720 (BW) to 0.23548 (JF). The observed heterozygosity of the FJ and MR populations was approximately half of the expected heterozygosity. The inbreeding coefficients of these two populations were relatively high, indicating significant inbreeding levels. The observed heterozygosity was much higher than the expected heterozygosity in the JX and QW populations, and the inbreeding coefficients of these two populations were negative, indicating that they are likely outbreeding. The nucleotide diversity of the *H*. *hainanensis* populations ranged from 0.00096 to 0.00138, with the KF population having the lowest genetic diversity and the MR, JF, FJ, and BW populations having a relatively higher genetic diversity (**Table 1**).

### 3.2 Population structure and genetic differentiation among populations

**Figure 1:**
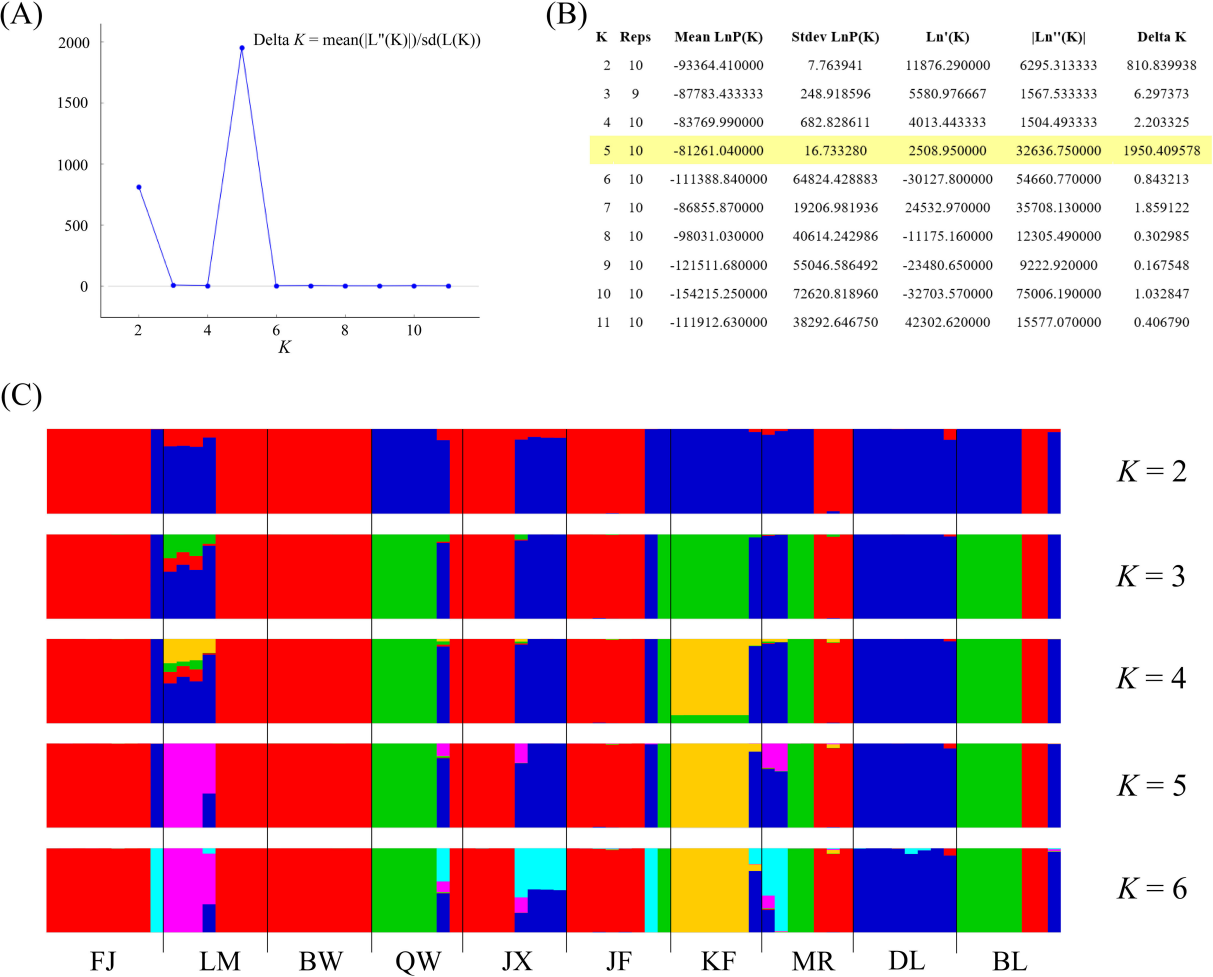
Results of the STRUCTURE analysis. **(A)** The number of clusters *K* was inferred to be 5 using the Δ*K* method proposed by Evanno et al. (2005). **(B)** Log probabilities and Δ*K* values for K from 2 to 11. **(C)** The results of individual assignment at five different *K* (from 2 to 6). Each vertical bar represents an individual, and the proportion of the colors corresponds to the posterior probability of assignment to one of *K* genetic clusters.

**Figure 2:**
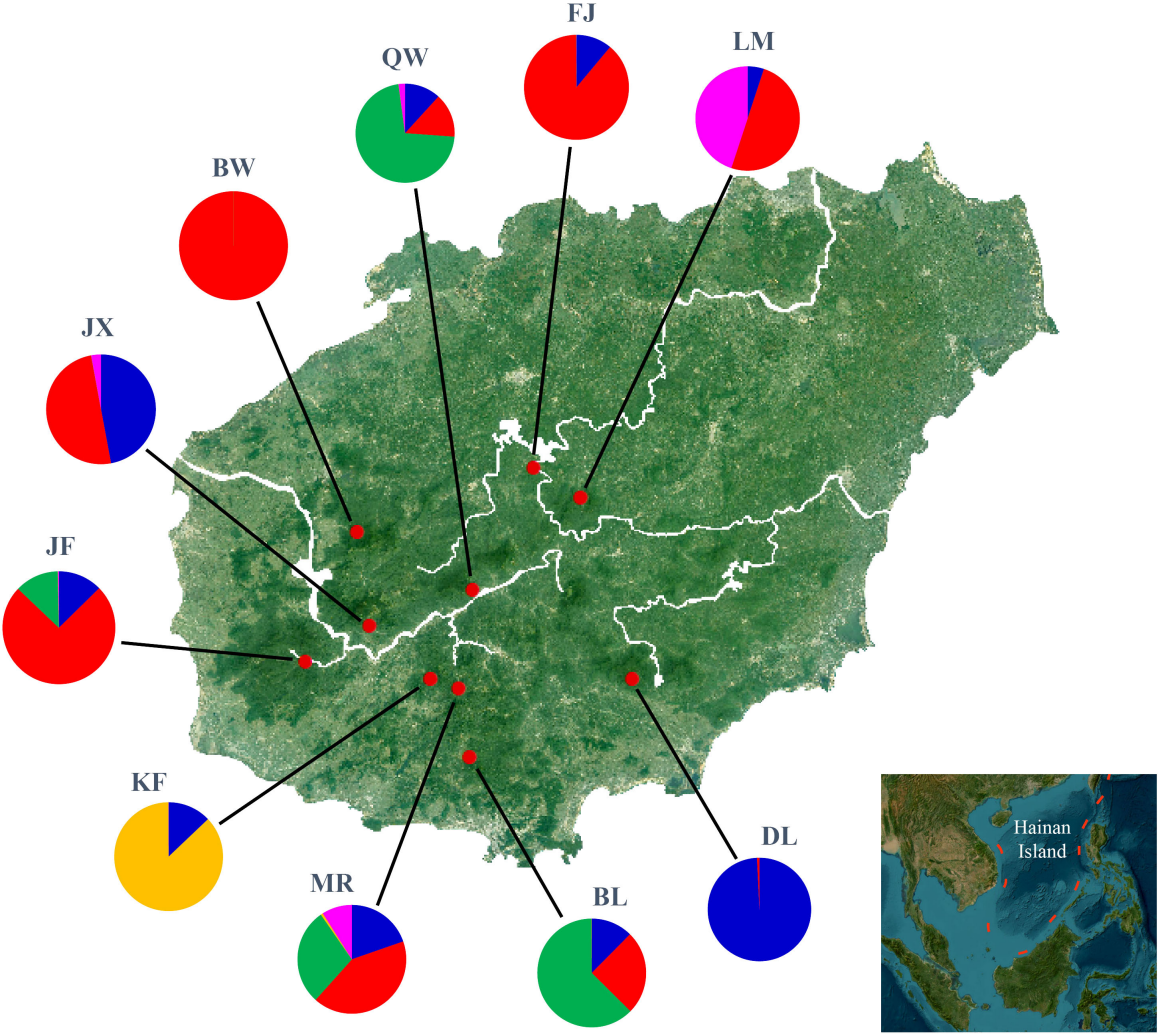
Geographic location of *Hopea hainanensis* populations sampled in this study (red dots). Pie charts illustrate the proportion of each of the five genetic clusters identified by STRUCTURE analyses for each population.

**Figure 3:**
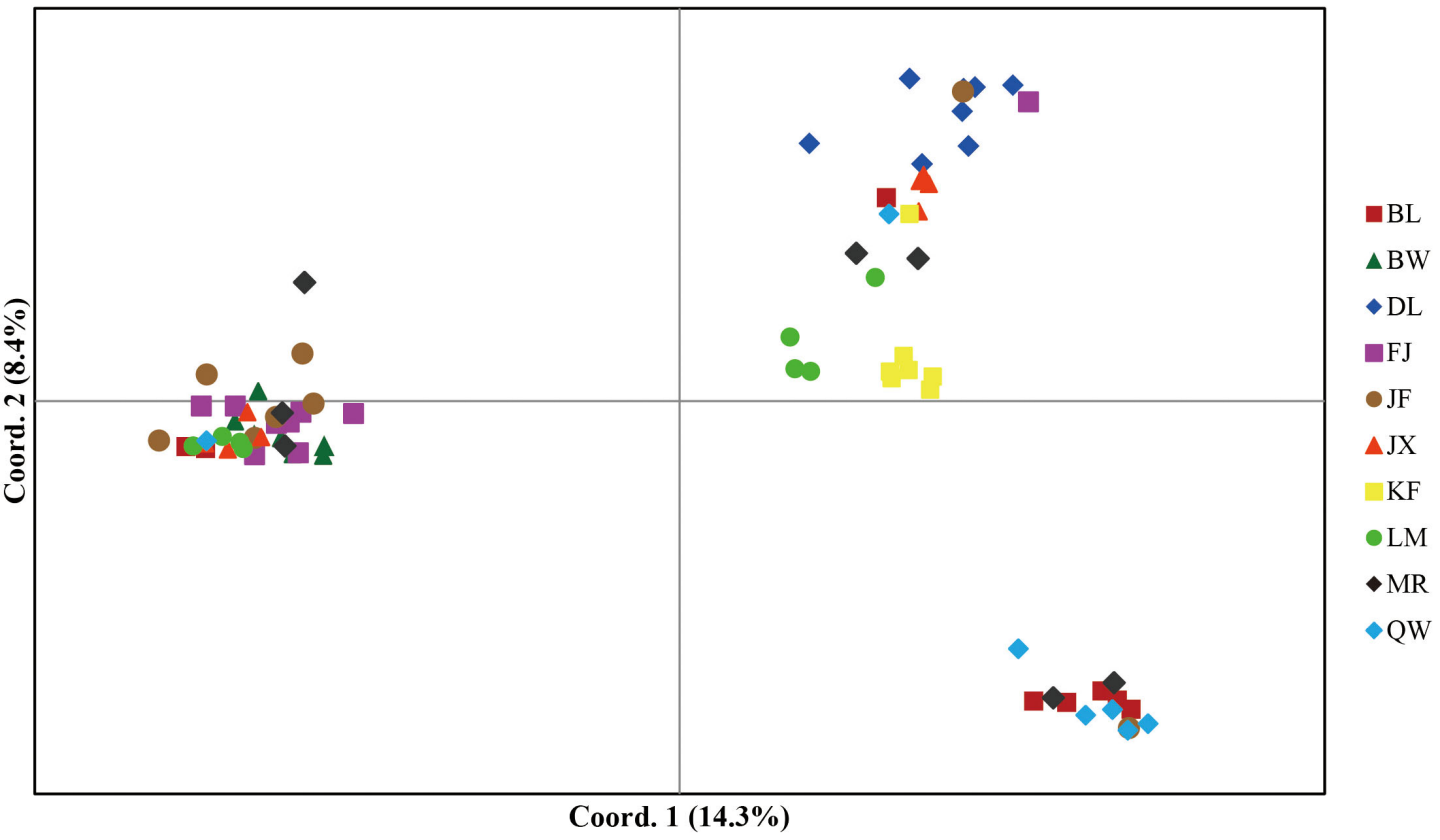
Principal component analysis (PCA) of individual samples from the *H*. *hainanensis* populations. The amount of variance explained by each component is indicated on PC1 and PC2 axes. Distinct colors and shapes represent individuals of different geographic origins.

In model-based STRUCTURE analysis, the maximum value of Ln(*K*) was achieved when *K* = 5. Following the method developed by Evanno et al. (2005), *K* = 5 was found to best fit the data, indicating five distinct genetic clusters within the *H*. *hainanensis* populations on Hainan Island (**Figure 1**). There was no apparent geographic structure among the five genetic clusters (**Figure 2**). The genetic clusters colored in red and blue were geographically widespread and found in most of the sampled populations. The other three were more population specific, detected in 1 to 4 populations. The BW and DL populations were dominated by one genetic cluster, whereas the rest had at least two clusters. Only a few individuals showed mixed membership in the STRUCTURE analysis. Based on the first two principal components (PC1 and PC2), the *H*. *hainanensis* samples collected from Hainan Island were classified into three discrete genetic groups, each containing individuals from multiple populations (**Figure 3**). Except for the BW, DL, and FK populations, individuals sampled from the same population were assigned to at least two different genetic groups (**Figure 3**). The NJ tree reconstructed using the SNP data showed a similar pattern of individual clusters to that identified in the STRUCTURE analysis (**Supplementary Figure S1**).

A pairwise comparison of genetic differentiation showed that Wrighten *F*_st_ ranged from 0.0321, between the BL and QW populations, to 0.1814, between the BW and DL populations, with a mean of 0.0975, indicating low to moderate differentiation among *H*. *hainanensis* populations (**Table 2**). The *F*-statistics, calculated by AMOVA, was 0.1367, suggesting that the overall differentiation is moderate (**Supplementary Table S2**). When intra- and inter-population levels were considered in AMOVA, 86.33% of the total molecular variation was found to be partitioned within populations. Isolation-by-distance, i.e., a significant linear correlation between genetic divergence and geographic distance, could not be detected by Mantel’s test (**Supplementary** **Figure S2**).

**Table 2:** Pairwise comparison of the genetic differentiation between populations measured by *F*_st_.

	BL	BW	DL	FJ	JF	JX	KF	LM	MR	QW
BL		0.106426	0.128166	0.106411	0.066407	0.075980	0.113281	0.080823	0.061390	0.032097
BW			0.181353	0.057191	0.062590	0.083326	0.163143	0.078393	0.095148	0.127676
DL				0.172434	0.129984	0.113467	0.151688	0.137713	0.120300	0.133270
FJ					0.052953	0.069807	0.159265	0.073314	0.081180	0.119384
JF						0.053057	0.118488	0.059931	0.063274	0.074939
JX							0.115707	0.062044	0.059894	0.078807
KF								0.122776	0.127102	0.111446
LM									0.067560	0.086638
MR										0.053428

### 3.3 Historical demography

**Figure 4:**
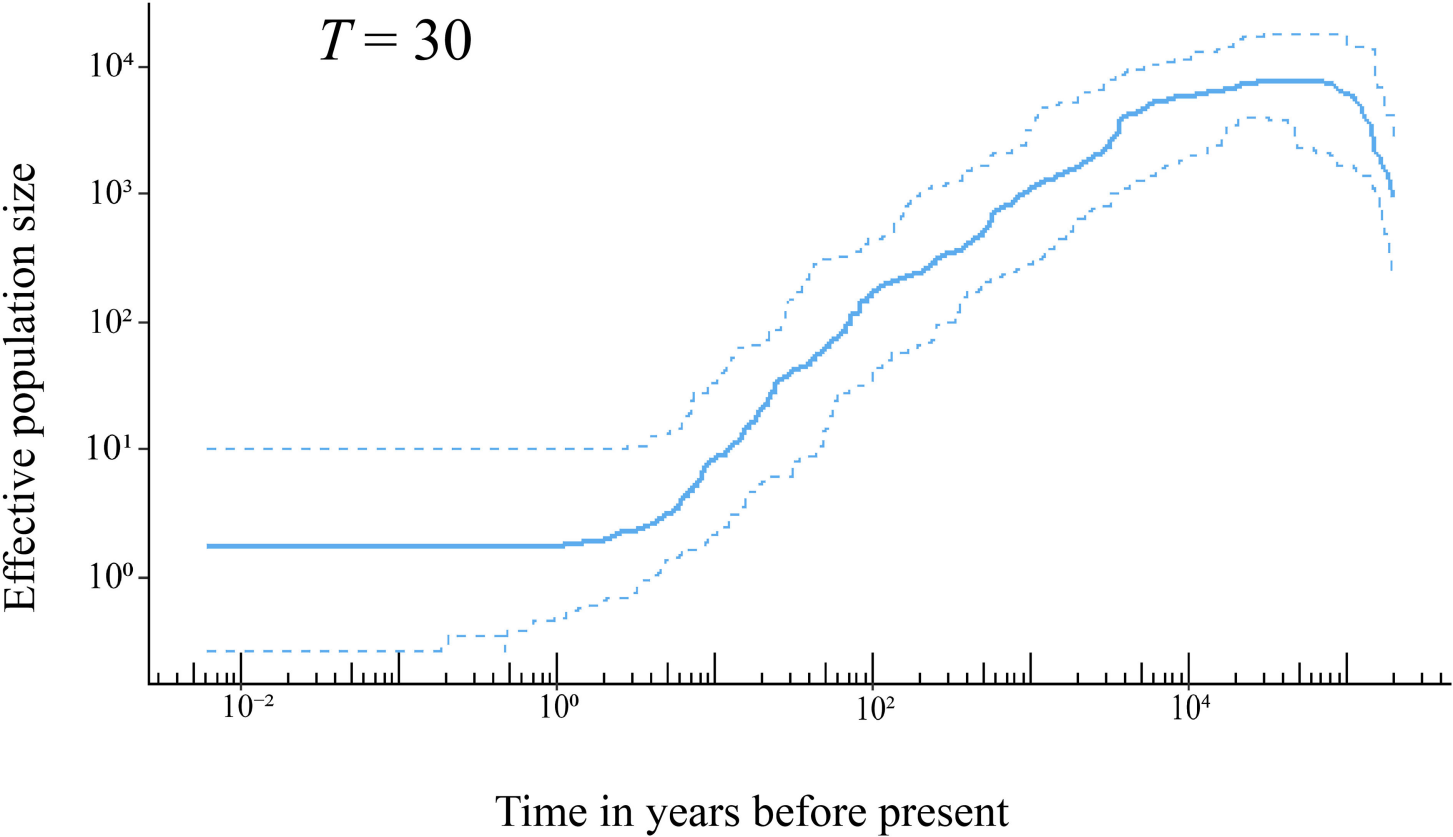
The demographic history of *H*. *hainanensis* inferred using stairway plot 2 with pooled individuals from the sampled populations. Thirty years was employed as the generation time of this species based on field observation. The solid line represents the estimation of the effective population size, with the two dotted lines delineating the 95% confidence interval of the estimation.

The results of stairway plot analysis indicated that the population of *H*. *hainanensis* on Hainan Island began to shrink approximately 20,000 years ago from an initial population size of approximately 10,000 individuals (**Figure 4**). The decline of the *H*. *hainanensis* population accelerated approximately 4,000 years ago and continued to the present, resulting in a small remaining population. Variations in generation time only slightly influenced the onset of population decline and size of the ancestral population before the decrease (**Supplementary Figure S3**).

## 4 Discussion

### 4.1 Low genetic diversity of *H*. *hainanensis* due to a severe population decline

Genetic diversities are commonly assessed for endangered dipterocarps and those that are predominant in Asian rainforest communities. Moderate to high levels of genetic variation within populations and weak differentiation among populations have been reported in some studies, e.g., Tito de Morais et al. (2015); Ghazoul (2016); Utomo et al. (2018), and Ng et al. (2019). Using 12 simple sequence repeat markers, Wang et al. (2020) found that heterozygosity, the number of alleles, and the proportions of low-frequency alleles in the endangered *H*. *hainanensis* were significantly lower than the non-endangered *H. dryobalanoides* of the same genus. As low-frequency alleles are more susceptible to loss than common alleles during population bottlenecks (Luikart et al., 1998), Wang et al. (2020) proposed that *H*. *hainanensis* on Hainan Island might have recently undergone such an event. This likely resulted in the loss of low-frequency alleles and a significant reduction in genetic diversity.

In this study, using RADseq, the nucleotide diversity (π) of *H*. *hainanensis* was shown to range from 0.96 × 10^−3^ to 1.38 × 10^−3^, with an average of 1.17 × 10^−3^ (**Table 1**). The genetic variation of *H*. *reticulata*, another species of the genus *Hopea* in Hainan Island, has been studied recently using RADseq, yielding an average nucleotide diversity of 0.91 × 10^−3^, slightly lower than that of *H*. *hainanensis* (Tang et al., 2024). The limited geographic distribution and severe population decline have been suggested as reasons for the low genetic variation observed in *H*. *reticulata*. However, the nucleotide diversity of *H*. *hainanensis* is significantly lower than several other PSESPs. For example, *Acer yangbiense*, a PSESP native to northwest Yunnan, showed a nucleotide diversity ranging from 2.54 × 10^−3^ to 3.41 × 10^−3^ based on whole-genome resequencing of 105 individuals from 10 populations (Ma et al., 2022). Only 31 individuals from the two wild populations of *R. griersonianum*, another PSESP in Yunnan, yielded an average π of 1.94 × 10^−3^, based on whole-genome resequencing (Ma et al., 2021). Furthermore, first-class protected wild plants, such as *Ginkgo biloba* (i = 2.19 × 10^−3^–2.41 × 10^−3^, Zhao et al., 2019), *Thuja sutchuenensis* (u = 2.19 × 10^−3^, Qin et al., 2021), and *Cathaya argyrophylla* (π = 2.10 × 10^−3^, Wang and Ge, 2006), also exhibit higher nucleotide diversities than *H*. *hainanensis*. Population genetic theory suggests that effective population size is positively related to the amount of genetic variation maintained in a population (Charlesworth, 2009; Ellegren and Galtier, 2016). A reduction in population size and demographic bottlenecks have been generally observed in endangered species. For instance, there have been at least two bottleneck events in *A*. *yangbiense* (Ma et al., 2022) and three significant bottlenecks in *R*. *griersonianum* (Ma et al., 2021) and *G*. *biloba* (Zhao et al., 2019). As with *H*. *reticulata*, a significant decline in the *H*. *hainanensis* population on Hainan Island over the last 20,000 years has been demonstrated. In conclusion, both the small effective population size and low genetic diversity in *H*. *hainanensis* are attributable to a severe population contraction in this species.

The shrinking of the *H*. *hainanensis* population on Hainan Island may have been induced by rising sea levels following the Last Glacial Maximum (LGM) [26.5–19 × 10^3^ years ago (Clark et al., 2009)]. During the LGM, the landmass of Sundaland expanded due to lower sea levels, and environmental conditions were indicated to be suitable for Dipterocarpaceae (Raes et al., 2014). The rainforests of Sundaland covered a substantially larger area than they do at present (Cannon et al., 2009). Following the end of the LGM, the low-altitude areas of Sundaland gradually submerged into the sea due to rising sea levels. Approximately 38.5% of the lowland rainforests in Sundaland disappeared compared with the LGM period (Cannon et al., 2009). Hainan Island, once on the edge of the landmass in the north South China Sea, was eventually separated from the mainland, with its land area reduced because of the rising sea levels after the LGM (Xiong et al., 2020). Stairway plot analysis indicated that the decline in the *H*. *hainanensis* population began approximately 20 × 10^3^ years ago, aligning well with the timing of the end of the LGM (**Figure 4**). Consequently, the rising sea levels after the LGM likely led to the contraction of the *H*. *hainanensis* population on Hainan Island.

Deforestation and excessive logging have also contributed to the reduction in the *H*. *hainanensis* population. Trees from the Dipterocarpaceae family are a well-known resource for timber production. Ly et al. (2018) estimated that approximately 50% to 70% of the *H*. *hainanensis* population has been cut down over the past 300 years. The high-quality wood produced from this species is suitable for building boats, bridges, and houses and making furniture. It is likely that *H*. *hainanensis* has been logged since human activity commenced on Hainan Island, approximately 7,000 to 3,000 years ago (Pan, 1999). Moreover, deforestation accelerated greatly in the 20th century, with approximately 80 to 95% of the primary forest being destroyed or converted into rubber or eucalyptus plantations on the Island (Zhou et al., 2005; Lin et al., 2017). The stairway plot showed an accelerated reduction in the *H*. *hainanensis* population over the past 100 years, in line with the intensified deforestation and logging during the 20th century on Hainan Island. This indicates that human disturbance is a significant factor exacerbating the decline in the *H*. *hainanensis* population.

It is worth noting that Chen et al. (2022) also assessed the genetic diversity and population structure of *H*. *hainanensis* on Hainan Island using RADseq. Nonetheless, there are several important differences between Chen et al. (2022) and the current study. First, we sampled 78 individuals from 10 populations, whereas Chen et al. (2022) collected 47 samples from 7 populations. Four populations—FJ, KF, BL, and MR—included in the current study were not represented in Chen et al. (2022). Notably, the KF population was characterized by a population-specific genetic cluster (**Figure 2**), and the MR population had the highest level of nucleotide diversity among the 10 sampled populations (**Table 1**). Extensive geographic sampling lays the foundation for a comprehensive understanding of the pattern of genetic variation in *H*. *hainanensis*. Differences in population sampling may contribute to the differences in genetic structure inferred by the two studies. In our STRUCTURE analysis, the best *k* was 5, whereas Chen et al. (2022) identified it as 2. Knowledge of the number and geographic distribution of genetic clusters is essential for accurately recognizing populations that require priority in conservation. Underestimation of genetic clusters may cause a loss of variation due to the misidentification of populations that need conservation. Finally, by performing stairway plot analysis, we demonstrated a severe and persistent decline in the *H*. *hainanensis* population on Hainan Island (**Figure 4**) and discussed potential reasons for the population size contraction. However, historical demography was not addressed in Chen et al. (2022). In conclusion, based on broader geographic population sampling, we studied the genetic diversity, population structure, and demographic history of *H*. *hainanensis* on Hainan Island, which could shed new light on the pattern of genetic variation and demographic history of *H*. *hainanensis* on this Island.

### 4.2 Genetic differentiation among *H*. *hainanensis* populations

Before the rise in sea levels and human colonization, *H*. *hainanensis* likely had a wider distribution on Hainan Island than it does today. Populations of *H*. *hainanensis* were probably connected through gene flows mediated by seed and pollen dispersal. Typically, the dispersal distance of seeds from the Dipterocarpaceae family is usually within 100 m (Suzuki and Ashton, 1996; Smith et al., 2015). Seeds may be carried by storms and transferred as much as several hundred meters from their parent trees (Ghazoul, 2016). Dipterocarps with winged fruits could realize longer distance of seed dispersal through autorotation when falling than those with wingless fruits (Seidler and Plotkin, 2006). Pollen-mediated gene flow for species in this family has been estimated to extend from tens to more than three hundreds of meters (Widiyatno et al., 2017). Notably, long-distance pollen flows have been reported in *Neobalanocarpus heimii*, *Dipterocarpus tempheses*, and several *Shorea* species (Konuma et al., 2000; Kenta et al., 2004; Widiyatno et al., 2017). Using paternity analysis, the average distance of pollen flow in *N*. *heimii* was estimated to be 191 m, with several pollination events exceeding 400 m (Konuma et al., 2000). Owing to the potential for the long-distance dispersal of pollen and seeds, weak differentiation among populations with continuous distribution is commonly observed in dipterocarps (Lee et al., 2000; Lim et al., 2002; Ohtani et al., 2021; Mishra et al., 2023). Consequently, we might expect a low level of differentiation among Hainan Island’s *H*. *hainanensis* populations because of gene flows among them prior to habitat fragmentation and population contraction.

With rising sea levels and increased logging activities, the populations of *H*. *hainanensis* on Hainan Island became gradually fragmented and diminished in both size and distribution area. According to population genetic theory, small and isolated *H*. *hainanensis* populations should increasingly diverge from each other due to intensified genetic drift (Hartl and Clark, 2007). Consistent with theoretical expectations, moderate levels of differentiation between some geographically distant populations of *H*. *hainanensis* were detected (**Table 2**). The genetic clusters identified in this species are generally widely distributed in the lowland rainforests of Hainan Island, suggesting potential gene flow among populations. This could explain the lack of detectable geographic structure in SNP variations within this species (**Supplementary Figure S2**). After population fragmentation and contraction, different populations probably retained different alleles due to random genetic drift, driving divergence among them (**Figure 2**). In conclusion, the increased differentiation between *H*. *hainanensis* populations is likely the result of genetic drift in small and isolated populations, as well as interrupted gene flow caused by habitat fragmentation and population contraction.

### 4.3 Conservation implication

Genetic diversity is crucial for a species to adapt to changing environments and ensure long-term survival. The genetic diversity of *H*. *hainanensis*, assessed using genome-wide SNP variation, is significantly lower than several other studied endangered species and species with extremely small populations. The key to conserving *H*. *hainanensis* lies in expanding its population size and restoring its genetic diversity. Populations such as JF and MR, which exhibit the highest levels of nucleotide diversity, and JF, MR, QW, and BL, which harbor diverse genetic clusters, should be prioritized for conservation efforts. These populations could be served as a provenance to cultivate saplings and young trees utilized in the restoration of *H*. *hainanensis* populations. Assisting the growth of seedlings into the sapling stage is an effective method for promoting population growth. Implementing the above recommendation in conservation activities would prevent a further loss of genetic diversity in this species, gradually restore wild populations, and thereby enhance the integrity and ecological services of the lowland rainforest ecosystem on Hainan Island.

## Data Availability

The datasets generated during the current study are available inthe National Center for Biotechnology Information (NCBI) SequenceRead Archive (SRA) under accession number PRJNA1083891, https://www.ncbi.nlm.nih.gov/sra/PRJNA1083891.
