# Supplementary material for: Conservation genomic study of Hopea hainanensis (Dipterocarpaceae), an endangered tree with extremely small populations on Hainan Island, China
Source: Front Plant Sci. 2024 Sep 4;15:1442807. doi: 10.3389/fpls.2024.1442807 (PMC11408178; doi:10.3389/fpls.2024.1442807)
Supplement: Supplementary file 1 [file DataSheet1.docx]

Supplementary Material

**Conservation genomic study of *Hopea hainanensis* (Dipterocarpaceae), an endangered tree with extremely small populations on Hainan Island, China**

Tang Liang^1,3* †^, Long Jun-qiao^2 †^, Wang Hai-ying^3^, Rao Chao-kang^3^, Long Wen-xing^4^, Yan Li^2*^, Liu Yong-bo^5*^

*** Correspondence:** Tang Liang: [tangliang@hainanu.edu.cn](mailto:tangliang@hainanu.edu.cn); Yan Li: [yanli@mail.cgs.gov.cn](mailto:yanli@mail.cgs.gov.cn%20); Liu Yong-bo: [liuyb@craes.org.cn](mailto:liuyb@craes.org.cn)

## Supplementary Figures


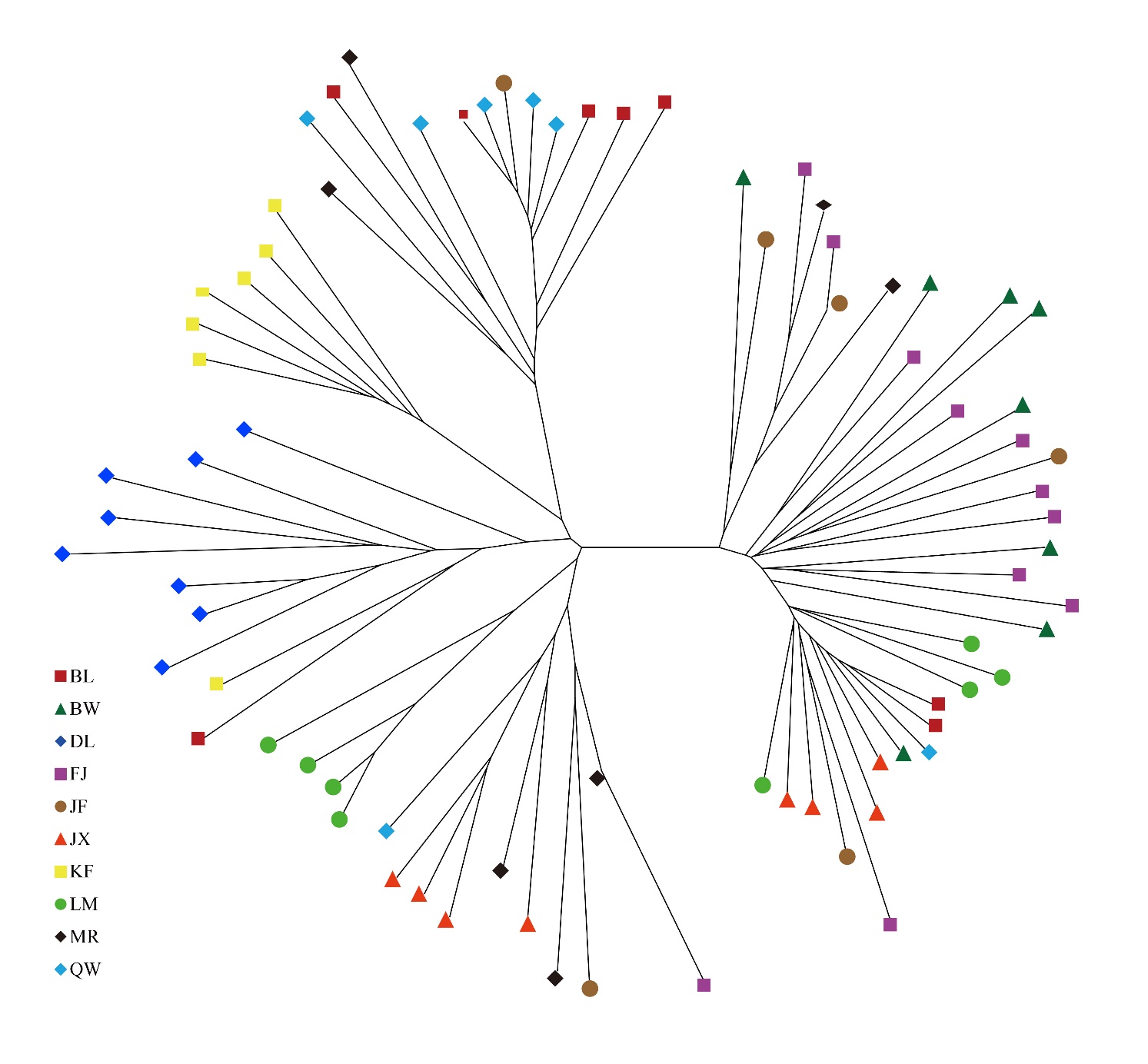


**Supplementary Figure 1.** Neighbor joining tree reconstructed using individual *H*. *hainanensis* samples based on a *p*-distance matrix inferred from SNP sites.


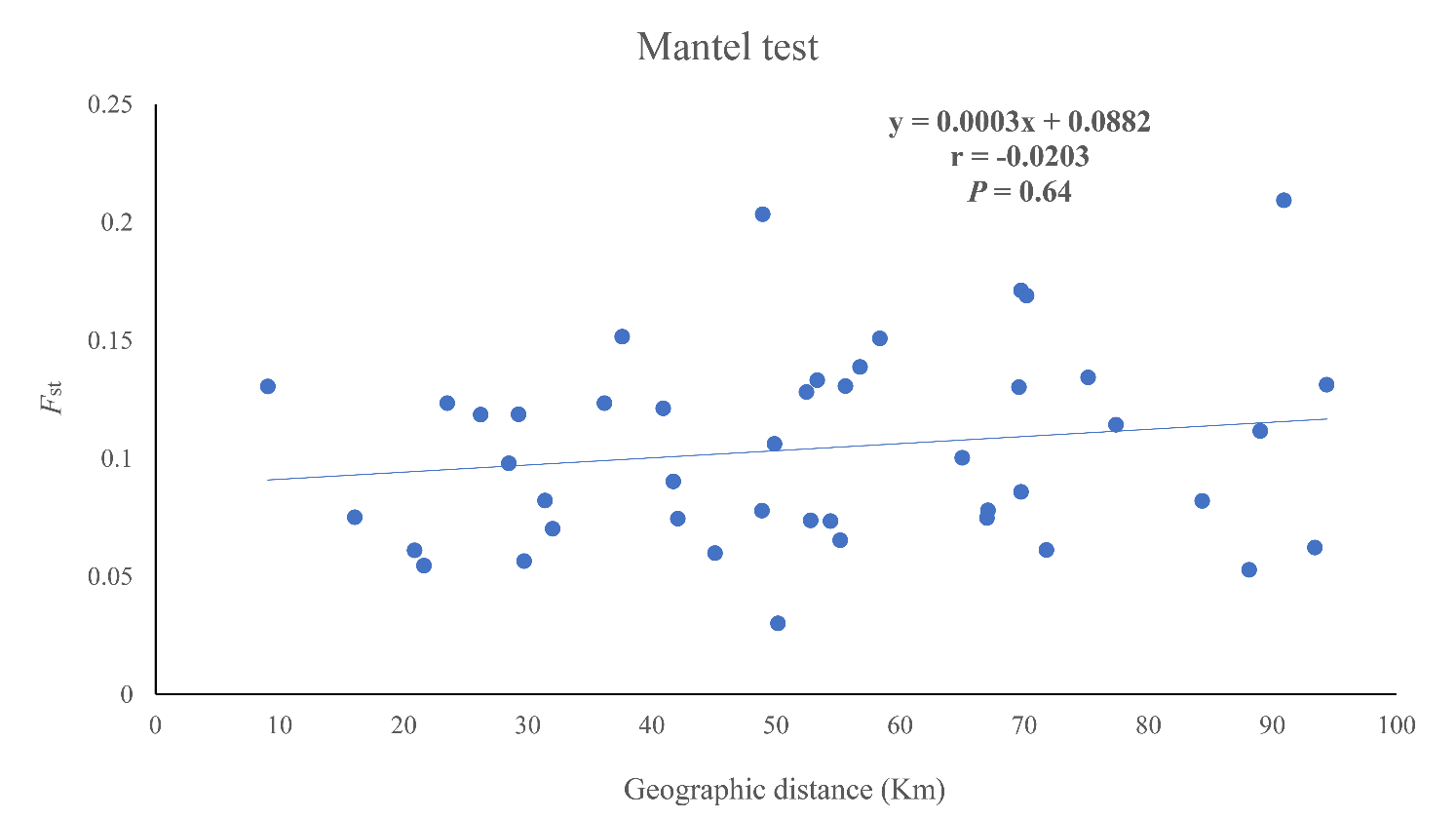


**Supplementary Figure 2.** Mantel test of the correlation between genetic distance (Wright’s *F*_st_) and geographical distance (km).


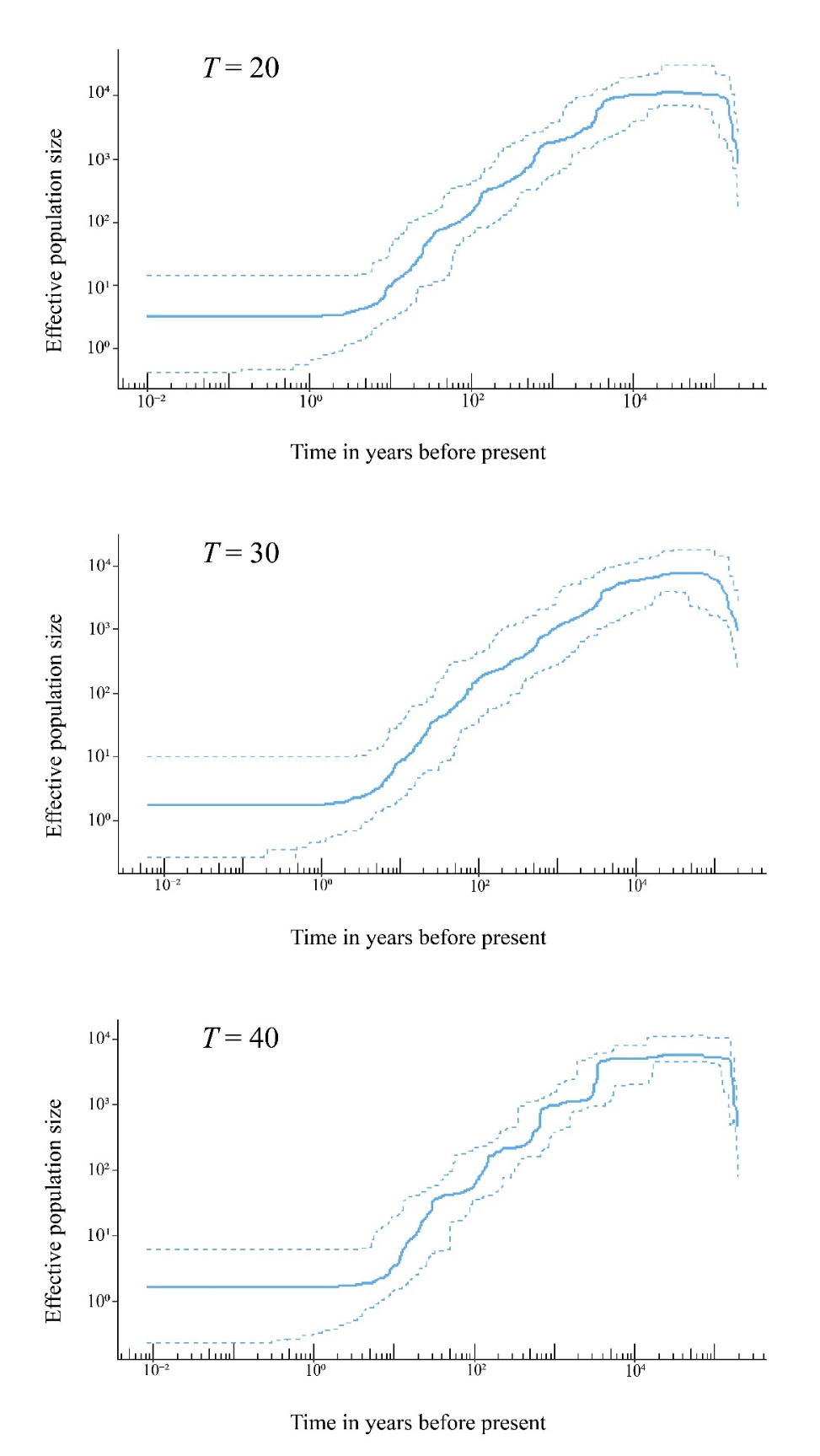


**Supplementary Figure 3**. The demographic history of *H*. *hainanensis* inferred using Stairway plot 2, based on pooled individuals from the 10 sampled populations. The generation time was varied as (A) 20, (B) 30, (C) 40 years while other parameters were kept the same. The solid line represents the estimation of effective population size, with the two dotted lines delineating the 95% confidence interval of the estimation.

## Supplementary Tables

**Supplementary Table 1.** The numbers of reads and nucleotides for each individual samples

| Population | Individuals | No. of clean reads | No. of clean nucleotides |
| --- | --- | --- | --- |
| BL | BL1.fq.gz | 3,520,104 | 528,015,600 |
|  | BL2.fq.gz | 1,090,432 | 163,564,800 |
|  | BL3.fq.gz | 3,595,212 | 539,281,800 |
|  | BL4.fq.gz | 4,109,410 | 616,411,500 |
|  | BL5.fq.gz | 2,483,736 | 372,560,400 |
|  | BL6.fq.gz | 3,060,394 | 459,059,100 |
|  | BL7.fq.gz | 1,531,222 | 229,683,300 |
|  | BL8.fq.gz | 2,122,728 | 318,409,200 |
| BW | BW1.fq.gz | 241,552 | 36,232,800 |
|  | BW2.fq.gz | 980,334 | 147,050,100 |
|  | BW3.fq.gz | 955,370 | 143,305,500 |
|  | BW4.fq.gz | 1,545,462 | 231,819,300 |
|  | BW5.fq.gz | 894,504 | 134,175,600 |
|  | BW6.fq.gz | 629,226 | 94,383,900 |
|  | BW7.fq.gz | 3,357,534 | 503,630,100 |
|  | BW8.fq.gz | 1,160,930 | 174,139,500 |
| DL | DL1.fq.gz | 10,521,382 | 1,578,207,300 |
|  | DL2.fq.gz | 1,055,692 | 158,353,800 |
|  | DL3.fq.gz | 1,464,666 | 219,699,900 |
|  | DL4.fq.gz | 3,961,028 | 594,154,200 |
|  | DL5.fq.gz | 1,603,624 | 240,543,600 |
|  | DL6.fq.gz | 3,526,434 | 528,965,100 |
|  | DL7.fq.gz | 2,166,034 | 324,905,100 |
|  | DL8.fq.gz | 1,310,872 | 196,630,800 |
| FJ | FJ1.fq.gz | 7,503,062 | 1,125,459,300 |
|  | FJ2.fq.gz | 2,924,338 | 438,650,700 |
|  | FJ3.fq.gz | 4,456,782 | 668,517,300 |
|  | FJ4.fq.gz | 4,331,190 | 649,678,500 |
|  | FJ5.fq.gz | 3,171,390 | 475,708,500 |
|  | FJ6.fq.gz | 5,213,420 | 782,013,000 |
|  | FJ7.fq.gz | 370,610 | 55,591,500 |
|  | FJ8.fq.gz | 1,439,810 | 215,971,500 |
|  | FJ9.fq.gz | 12,302,576 | 1,845,386,400 |
| JF | JF1.fq.gz | 990,696 | 148,604,400 |
|  | JF3.fq.gz | 1,369,194 | 205,379,100 |
|  | JF4.fq.gz | 2,372,992 | 355,948,800 |
|  | JF5.fq.gz | 12,324,198 | 1,848,629,700 |
|  | JF6.fq.gz | 1,805,592 | 270,838,800 |
|  | JF7.fq.gz | 3,743,022 | 561,453,300 |
|  | JF8.fq.gz | 1,390,574 | 208,586,100 |
|  | JF9.fq.gz | 7,1695,14 | 1,075,427,100 |
| JX | JX1.fq.gz | 4,142,170 | 621,325,500 |
|  | JX2.fq.gz | 3,276,532 | 491,479,800 |
|  | JX3.fq.gz | 2,507,794 | 376,169,100 |
|  | JX4.fq.gz | 1,912,498 | 286,874,700 |
|  | JX5.fq.gz | 1,786,722 | 268,008,300 |
|  | JX6.fq.gz | 2,631,940 | 394,791,000 |
|  | JX7.fq.gz | 3,275,072 | 491,260,800 |
|  | JX8.fq.gz | 3,503,188 | 525,478,200 |
| KF | KF1.fq.gz | 4,357,656 | 653,648,400 |
|  | KF2.fq.gz | 4,020,654 | 603,098,100 |
|  | KF3.fq.gz | 2,424,092 | 363,613,800 |
|  | KF5.fq.gz | 2,510,702 | 376,605,300 |
|  | KF6.fq.gz | 1,809,248 | 271,387,200 |
|  | KF8.fq.gz | 1,717,118 | 257,567,700 |
|  | KF9.fq.gz | 2,380,450 | 357,067,500 |
| LM | LM1.fq.gz | 3,343,444 | 501,516,600 |
|  | LM2.fq.gz | 3,170,438 | 475,565,700 |
|  | LM4.fq.gz | 2,950,188 | 442,528,200 |
|  | LM5.fq.gz | 2,322,552 | 348,382,800 |
|  | LM6.fq.gz | 2,132,176 | 319,826,400 |
|  | LM7.fq.gz | 1,498,658 | 224,798,700 |
|  | LM8.fq.gz | 1,510,702 | 226,605,300 |
|  | LM9.fq.gz | 2,262,514 | 339,377,100 |
| MR | MR1.fq.gz | 3,684,610 | 552,691,500 |
|  | MR2.fq.gz | 4,157,752 | 623,662,800 |
|  | MR3.fq.gz | 8,811,812 | 1,321,771,800 |
|  | MR4.fq.gz | 9,884,438 | 1,482,665,700 |
|  | MR5.fq.gz | 2,523,868 | 378,580,200 |
|  | MR6.fq.gz | 2,745,090 | 411,763,500 |
|  | MR7.fq.gz | 4,928,838 | 739,325,700 |
| QW | QW1.fq.gz | 7,963,500 | 1,194,525,000 |
|  | QW2.fq.gz | 304,086 | 45,612,900 |
|  | QW3.fq.gz | 395,392 | 59,308,800 |
|  | QW4.fq.gz | 1,521,066 | 228,159,900 |
|  | QW5.fq.gz | 3,485,612 | 522,841,800 |
|  | QW6.fq.gz | 1,807,158 | 271,073,700 |
|  | QW7.fq.gz | 3,808,968 | 571,345,200 |

**Supplementary Table 2.** Analysis of molecular variance (AMOVA) for *H*. *hainanensis* populations at inter-population and intra-population levels.

| Source of variation | Sum of squares | Variance components | Percentage variation |
| --- | --- | --- | --- |
| Among populations | 55.799 | 0.318 | 13.675 |
| Within populations | 237.351 | 2.010 | 86.325 |
